# Novel ZrO_2_-glycine nanocomposite as eco-friendly high temperature corrosion inhibitor for mild steel in hydrochloric acid solution

**DOI:** 10.1038/s41598-022-13359-y

**Published:** 2022-06-03

**Authors:** Ruby Aslam, Mohammad Mobin, Mohd. Shoeb, Jeenat Aslam

**Affiliations:** 1 Corrosion Research Laboratory, Department of Applied Chemistry, Faculty of Engineering and Technology, Aligarh Muslim University, Aligarh, 202002 India; 2 Department of Chemistry, College of Science, Taibah University, Yanbu, Al-Madina 30799 Saudi Arabia

**Keywords:** Chemistry, Electrochemistry, Green chemistry, Materials chemistry

## Abstract

We report the green synthesis of novel ZrO_2_-Glycine nanocomposite referred to as ZrO_2_-Gly NC followed by its characterization using X-ray diffraction (XRD), Fourier transforms infrared (FT-IR) spectroscopy, SEM/EDX, and transmission electron microscopy (TEM) techniques. Further, the inhibition effect of the varying concentration of ZrO_2_-Gly NC on the corrosion of mild steel (MS) in 1 M HCl was investigated by weight loss and electrochemical measurements at 40–80 °C. The percentage inhibition efficacy of NC increased with the increase of concentration and temperature and reached about 81.01% at 500 ppm at 70 °C which decreased at 80 °C and exhibited 73.5% inhibition efficiencies. According to the polarization measurements, the investigated ZrO_2_-Gly NC works as a mixed-type inhibitor with predominantly inhibiting cathodic reaction. Also, the adsorption isotherm analysis indicated that the adsorption was spontaneous and followed the Langmuir adsorption isotherm. Furthermore, the contact angle measurement revealed the water-repelling property of the investigated inhibitor. The surface morphological study via SEM–EDS micrograph affirmed the appearance of a smooth surface in presence of inhibited media suggesting the formation of protective film by the adsorption of ZrO_2_-Gly NC on the surface of the MS even at higher temperature.

## Introduction

Pickling and cleaning/descaling operations often use acid-based solutions^1^. The main issue with using acid solutions is that they are excessively aggressive, resulting in an unwanted metal breakdown. Due to its excellent mechanical capabilities and low cost, mild steel (MS) is the most extensively used metal in a variety of industries, including petroleum, food, power generation, chemical, and electrochemical. As a result, increasing the lifespan of steel against corrosion has been a key challenge for research and industry, as corrosion causes significant metal loss and untimely catastrophic failure, resulting in high replacement costs and environmental issues. Corrosion causes significant financial losses every year all around the world. Corrosion expenses were predicted to be $2.5 trillion in 2015, accounting for 3.4% of world GDP. This figure excludes accidents, unexpected shutdowns, and environmental pollutants. Cathodic protection, coatings, and the use of corrosion inhibitors are all common procedures for reducing the risk of corrosion^2,3^. These solutions have a lot of advantages, but they also have certain disadvantages, therefore they motivate new research to improve anti-corrosion performance. Corrosion inhibitors can help extend the life of equipment in harsh environments, reduce the environmental, economic, health, and safety hazards associated with corrosion failures, and allow low-cost steel to be used instead of corrosion-resistant alloys^4^.

Literature study reveals that several reports are available describing the anticorrosion effect of amino acids^5,6^, biopolymers^7,8^, plant extracts^9,10^, rare earth metals^11^, organic compounds such as drugs^12,13^, surfactants^14^, and ionic liquids^15^ on metals and alloys. The main disadvantage of using plant materials as corrosion inhibitors is their instability; and readily biodegradability. Moreover, it is very difficult and tedious to isolate and purify them. More so, the preparation of plant extracts generally employs toxic solvents that can adversely affect the environment along with soil and aquatic lives after their discharge^16^. Most of these solvents are highly expensive and can adversely affect the economy of the extract preparation. Concerning the application of rare earth elements as corrosion inhibitors, their instability, and the extraction process involve the production of a lot of waste materials such as acids, ammonia, and some radioactive elements. This can potentially affect the environment if not properly treated. In the same way, the use of organic compounds is limited to their expensive and multi-step synthesis. Despite their nontoxicity, biodegradability, high solubility in aqueous media, relatively cheap and easy to produce with high purity, the use of amino acids is also limited^16^. Few authors noted a duplicate action of some amino acids on metal. Depending on operating conditions, such as pH solution or additive concentration, the amino acids can decrease the metal dissolution process (corrosion inhibitor) or increase it (corrosion accelerator). Therefore, using these compounds as metallic corrosion inhibitors should be accompanied by some precautions to avoid its corrosion catalytic effect. Moreover, the use of single amino acids generally needs a high amount of inhibitor usage^17^. One of the alternatives to decrease the inhibitor dosage is the infusion of certain inorganic substances to make composites^18,19^ which reduce the particle size thereby increasing the surface coverage and thus protecting metal against corrosion.

A significant effort at organic/inorganic nanocomposite (NC) materials has been focused on the ability to acquire the nanoscale structures via innovative synthetic methodologies. Synthesizing nanocomposites with an ability to control the corrosion on the metal surface leads to producing a sophisticated new generation of inhibitors with a higher corrosion inhibition efficiency, being cost-effective and ecofriendly. A study carried out by Kalajhai et al.^20^ on GO–Ag nanocomposites showed that the NC act as a nanobiocide to inhibit the sulphate reducing bacteria (SRB) and reduce the corrosion rate of X60 steel in seawater. The inhibition efficiency (IE) for 4, 7, 10, and 15 days of immersion was calculated as 79%, 96%, 98%, and 98%, respectively. Nanocomposites of aniline and CeO_2_ nanoparticles have been chemically synthesized by in-situ polymerization^21^. After examining structural evolutions and morphological characteristics of PANI/CeO_2_ nanocomposite (PCN) using various techniques such as XRD, FT-IR, XPS, SEM, and TEM analysis, the inhibition properties of synthesized PCN on mild steel (MS) corrosion in 0.5 M HCl were estimated using weight loss and electrochemical techniques. The results exhibited that the inhibition efficiency of PCN was found to increase almost linearly with concentration and 500 ppm of PCN showed 69.25% inhibition efficiencies. The morphological changes occurring on the metal surface were analyzed by ATR-IR, SEM/EDAX and AFM. Basik et al.^19^ reported that the silver cysteine-based-gold nanocomposite (Cys/Ag–Au NCz) acted as an efficient corrosion inhibitor. The maximum inhibition efficiency of 96% was observed at 303 K at 300 ppm. Cys/Ag–Au NCz affected both anodic and cathodic processes and its adsorption on steel surface followed the Langmuir adsorption isotherm. Solomon et al.^22^ prepared AgNPs/chitosan composite and tested their anti-corrosive behavior for St37 steel in 15% HCl solution at 25 and 60 °C temperature. The effectiveness and stability of glycine-functionalized graphene/Fe3O_4_ nanocomposite have been investigated by Aslam et al.^18^ for mild steel corrosion in 1 M HCl medium at 60 °C. A very low amount of compound i.e., 1 ppm was effective to mitigate corrosion of MS exhibiting 64.40% inhibition efficiency at 70 °C. At the same temperature, 100 ppm of NC exhibited 93.66% inhibition efficiency. Quadri et al.^23^ reported the synthesis and anti-corrosive applications of ZnO with poly(ethylene glycol,), poly-(vinylpyrrolidone) and polyacrylonitrile NC for MS in 5% HCl. At a maximum concentration of 1000 ppm, compounds exhibited the inhibition efficiencies as follows: ZnO/PVP (90.81%) > ZnO/PAN (81.72%) > ZnO/PEG (70.79%).

Keeping the above facts in mind we have synthesized the zerconium oxide nanoparticle (ZrO_2_ NP)/glycine NC. ZrO_2_ NPs have been chosen among transition metal oxide nanoparticles because of their unique electrical, thermal, catalytic, sensing, optical, mechanical, and biocompatible capabilities^24,25^. ZrO_2_ NPs have been widely used in a variety of applications, including bone implants, dental implants, photocatalysis, refractory materials, energy, fuel cells, gas sensors, solar cells, and seed germination^26^. Furthermore, due to their unique physicochemical features, ZrO_2_ NPs have antibacterial, antifungal, antioxidant, and anticancer effects^27^. Biological methods are employed to manufacture ZrO_2_ NPs in various morphologies and sizes from various sections of the plant, including the tuber, leaves, fruit, and flower. The advantage of green synthesis over chemical and physical methods is that it is a one-pot, clean synthesis that is environmentally friendly, cost-effective, quick, simple, and easily scaled up for large-scale nanoparticle syntheses, and it does not require the use of high temperatures, energy, pressure, or toxic chemicals. The bio compounds such as proteins, enzymes, polysaccharides, polyphenols, etc. present in the extract chelating with zirconium ions enhance the surface area and the number of active sites of ZrO_2_ nanoparticles. The higher surface area of ZrO_2_ nanoparticles can also facilitate a better favorable adsorption process. However, the of wider use nanoparticles is still impeded due to low yield, and problems in achieving small, uniform, and highly dispersed nanoparticles. Moreover, these nanostructured materials have some limitations arising from the tendency to aggregate because of their high specific area and strong interparticle interactions^28^. The solubility of NPs in water is also essential for their use in various industries to permit adequate distribution, and elimination and to render them biocompatible with reduced toxicity. To overcome this drawback, different strategies for the chemical stabilization of the NPs have been investigated, including the incorporation of polymer or amino acids on the NPs surface or the functionalization of the NPs surface.

In this context, an easy technique was used to synthesize a nanocomposite of ZrO_2_-Glycine (ZrO_2_-Gly NC). X-ray diffraction (XRD), Fourier transform infrared (FT-IR) spectroscopy, scanning electron microscopy/energy-dispersive X-ray spectroscopy (SEM/EDX), and transmission electron microscopy (TEM) techniques were used to analyze the produced ZrO_2_-Gly NC. Weight loss, PDP, EIS, contact angle measurement, and SEM–EDS experiments were used to investigate the anti-corrosive activity. The potential of corrosion inhibition of ZrO_2_-Gly NC has not been asserted until now, to the best of our knowledge.

## Experimental section

### Synthesis of ZrO_2_ nanoparticle

Green synthesis of ZrO_2_ NPs is a facile, swift, robust, one-pot synthesis and environmentally benign without the involvement of any mephitic and perilous chemical. With a sharp knife, the fresh bark sample was taken from the Eucalyptus globulus stem, rinsed well with deionized water, and dried thoroughly at 40 °C. The bark was dried and crushed into a fine powder. The Eucalyptus globulus extract was produced by placing 5 g of dry powder with 100 ml of DI water and heating it for 60 min at 70 °C on a magnetic stirrer. The extract was purified employing Whatman's filter paper, and the supernatant was extracted and kept in a refrigerator. Briefly, 20 ml of fresh extracted Eucalyptus bark extract (5 mg/ml) was cultivated in DI water and poured into 70 ml of aqueous zirconyl nitrate (ZrO(NO_3_)_2_) solution. The colloidal solution was obtained following 30 min of vigorous stirring at room temperature. Precipitation of the colloidal solution was accomplished by adding ammonia at a pH of 7.0, centrifuging at 8000 rpm for 10 min, and rinsing twice with DI water. The resultant solution is evaporated up to dryness in a vacuum oven and crushed into a fine powder using a mortar pestle. The powdered sample was sintered for three hours at 600 °C in a muffle furnace. The synthesized ZrO_2_ NPs were kept in a dry and dark condition till further utilization.

### Synthesis of ZrO_2_-Gly nanocomposite

**Figure 1:**
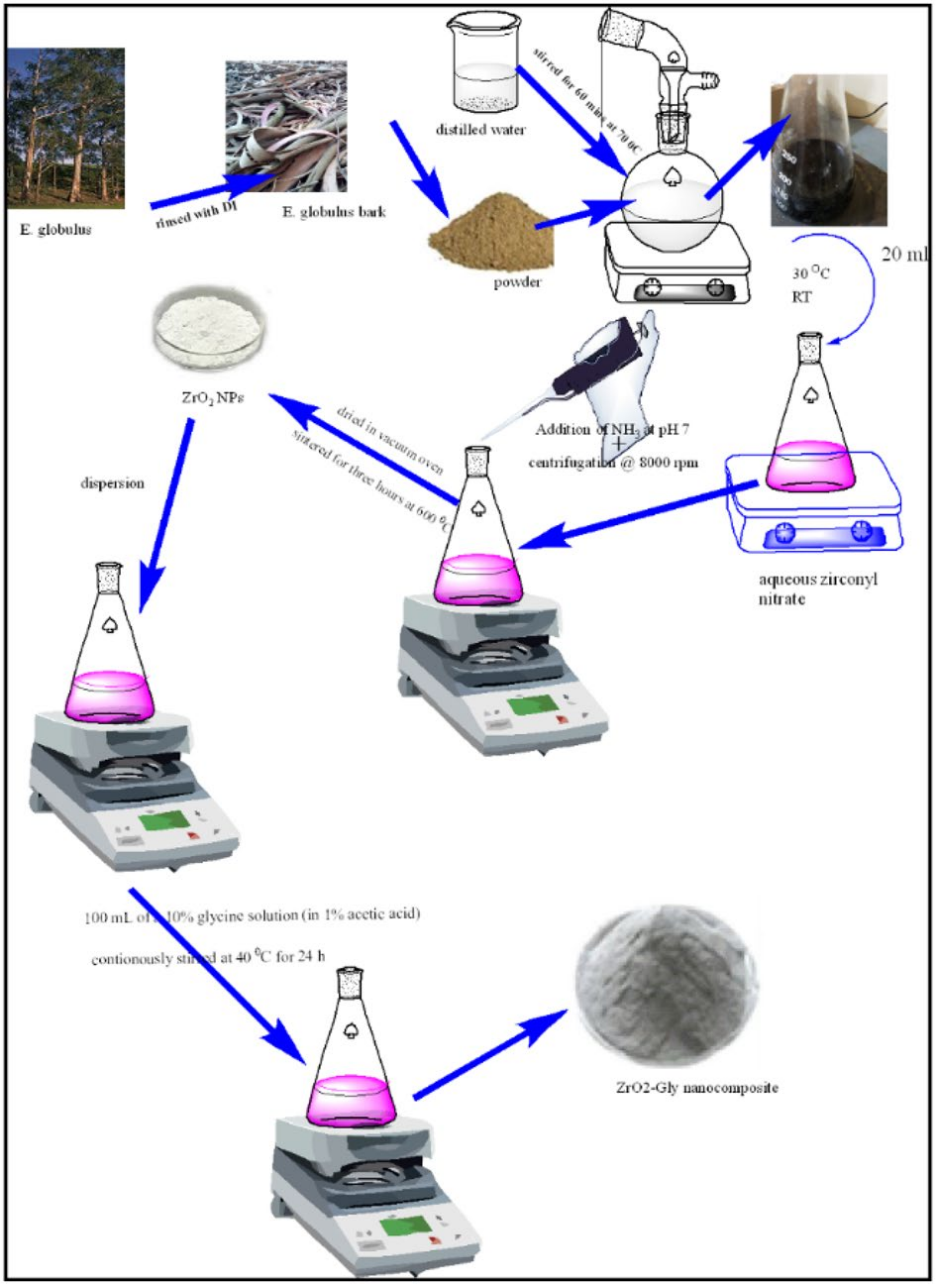
Synthesis of ZrO_2_-Gly nanocomposite.

Ultrasonication was used to functionalize the ZrO_2_NPs with glycine.The ZrO_2_ NPs (100 mg) dispersion in 50 ml distilled water was achieved employing ultrasonication for 30 min. The resulting suspension was then infused with 100 ml of a 10% glycine solution (in 1% acetic acid). Following that, the sample was continuously stirred for 24 h at 40 °C before being subjected to ultrasonication for another 120 min (Fig. 1). It was washed three times in distilled water to get rid of the extra glycine. The ZrO_2_-Gly nanocomposite was then dried. The ZrO_2_-Gly nanocomposite was further functionalized with glycine to enhance its dispersibility in aqueous environments.

### Corrosion studies

#### Materials and reagents

The working electrode (WE) in this experiment was mild steel and the composition is given in our earlier publications^29,30^. For weight loss measurement, the steel was cut into sheets with dimensions of 2.5 cm × 2 cm × 0.1 cm from a large strip. The WE were then polished with emery sheets in a range of grits (#320 to 1200) before being sonicated in 50% ethanol, dried, and stored in a desiccator. All corrosion experiments were carried out in 1 M HCl (Fisher Scientific, India), which was generated from HCl stock (37%, reagent grade).

#### Methods

Weight loss measurement was executed to study the anticorrosive impact of NC at 5 temperatures i.e., 40, 50, 60, 70, and 80 °C. The specimens were immersed in a 250 ml beaker containing the aggressive solution prepared without and with specific concentrations of NC for 6 h. After the soaking process, the steel specimens were removed from the analysis solution, corrosion product removed, cleaned properly using deionized water, dried, and weighed again precisely. All these tests were employed in triplicate and the standard deviation was for the three measurements has been given in the respective tables.

Three-electrode cells along with potentiostat/galvanostat supplied by Autolabwere used forthe conduction of the electrochemical measurements. The platinum wire, Ag/AgCl, and MS were utilized as reference, auxiliary and working electrodes, respectively. The polarization measurements with a constant potential sweep rate of 0.16 mV s^−1^ were recorded under potentiodynamic conditions within the range of -250 to + 250 mV. Impedance plots were also obtained by the automatic shift of potential from 10^−2^ to 10^5^ Hz with perturbation of 10 mV. To ensure suitable reproducibility each experiment was accomplished three times. Before each experiment, the working electrode was immersed for 30 min in the test solution to achieve a quasi-stable condition. All experiments were conducted at 40, 50 60, and 70 °C (within ± 1 °C) using a water bath to regulate the temperature of the cell. The measurements for each experimental condition were repeated thrice to confirm data reliability and reproducibility, and the average values were documented.

The corrosion protection efficiencies of NC by the PP and EIS method were predicted using the following formulas:

1 $$IE\left(\%\right)=\frac{{I}_{corr}^{o}-{I}_{corr}^{i}}{{I}_{corr}^{0}}\times 100$$

2 $$IE\left(\%\right)=\frac{{R}_{p}^{o}-{R}_{p}^{i}}{{R}_{p}^{o}}\times 100$$

where *I*_corr_^(0)^ and *I*_corr_^(i)^ are the corrosion current densities without and with the NC, *R*_p_^(0)^ and *R*_p_^(i)^ are the polarization resistances of MS without and with the NC, respectively.

Double-layer capacitance (C_dl_) is determined by means of the component of constant phase element (CPE) i.e., *Y*_0_ (proportional factor) and *n* (phase shift) following equation.

3 $${\text{C}}_{{{\text{dl}}}} = Y_{{0}} \left( {\omega_{\max } } \right)^{n - 1}$$

where *ω*_max_ = 2*πf*_max_ (*f*_max_ refers to the maximum frequency at which the imaginary part of the impedance has a maximum value).

The FT-IR spectra for synthesized NC and the adsorbed NC on the MS surface after 6 h immersion in 1 M HCl was obtained. The film was scrapped, combined with KBr, and converted into pellets for the adsorbed Fe- NC inhibitor spectrum.

With the aid of KRUSS GmbH Germany (model FM41Mk2 Easy drop) wettability analyzer, the wetting properties of the tested coupons were investigated to determine the hydrophobicity of the optimum concentration of NC relative to corroded WE. Furthermore, for morphological tests, the MS samples were subjected to aggressive media (1 M HCl) in the absence and presence of NC for 6 h and then were washed with distilled water and dried, and then analyzed by SEM/EDX. SEM instrument (model: JEOL JSM-6510LV) attached with EDX of model INCA, oxford was used for this purpose.

## Results and discussion

### Characterization of the synthesized NC

**Figure 2:**
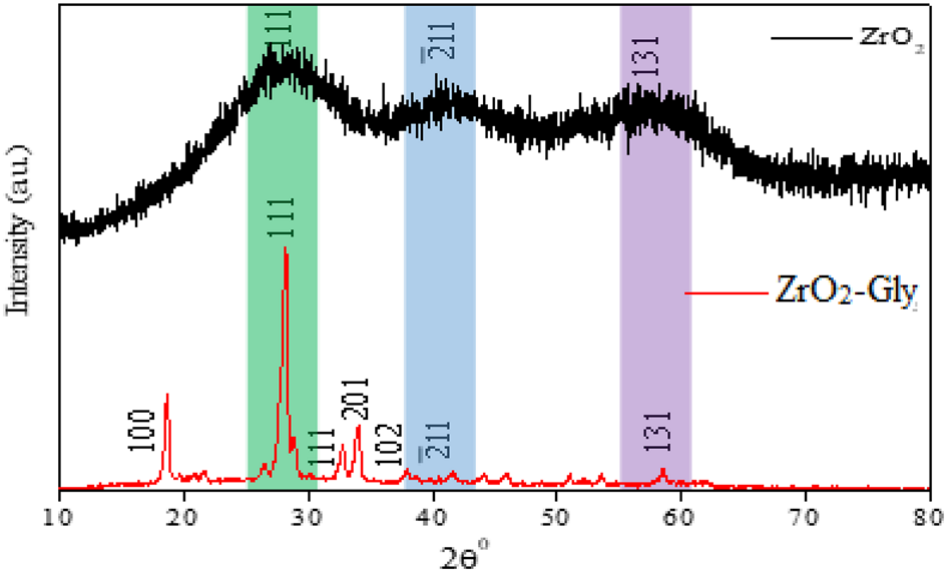
XRD pattern of ZrO_2_ NPs and ZrO_2_-Gly nanocomposite.

The XRD pattern of ZrO_2_-Gly NC and ZrO_2_ NPs is shown in Fig. 2. The XRD pattern of ZrO2 NPs showed three prominent peaks that confirmed its monoclinic structure, 2θ peak at 28.920, 41.720, 58.920 with miller indices (111), (− 211), and (131) respectively. After functionalization, of ZrO_2_ nanoparticles with glycine, seven well-resolved peaks were found in the data that correlate to the monoclinic structure crystal planes (100), (111), (111), (201), (102), (− 211), and (131) of monoclinic structure (JCPDS 37–1484), at 2θ = 18.50°, 29.00°, 32.61°, 34.02°, 37.04°, 42.06° and 59.53° respectively. Data clearly showed the successfully functionalized glycine with ZrO_2_ nanoparticles, a redshift on the signature peak of ZrO_2_ nanoparticles, i.e., 28.92° at (111) shifts to 29.00°.

**Figure 3:**
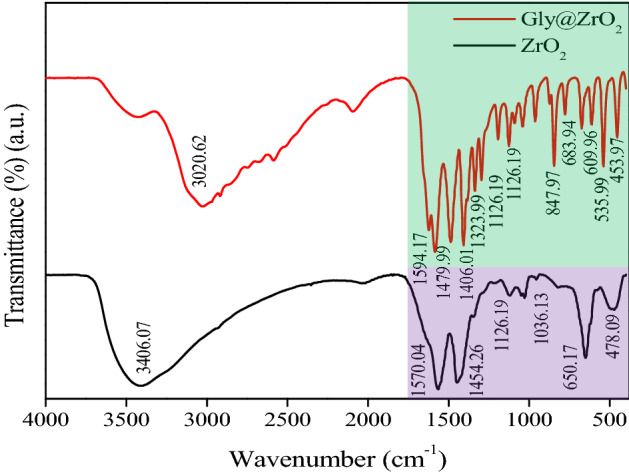
FT-IR spectra of ZrO_2_ NPs and ZrO_2_-Gly nanocomposite.

FT-IR analyses were performed to determine the attachment of glycine to the surface of the ZrO_2_ nanoparticle. Figure 3 illustrates the FT-IR spectra of ZrO_2_-Gly and ZrO_2_ nanoparticles in the 4000 cm^−1^ to 400 cm^−1^ range. FT-IR spectra of ZrO_2_ NPs (indicate with black line) showed the presence of Zr-O bond peaks at 478.09 cm^−1^ and 650.17 cm^−1^, as well as the presence of various peaks at 1036.13 cm^−1^, 1126.19 cm^−1^, 1454.26 cm^−1^, 1570.04 cm^−1^, and 3406.07 cm^−1^, which correspond to water OH bending vibration, CH stretching and bending vibration, and C–O stretching vibration from the EB extract. The FT-IR spectrum of ZrO_2_-Gly NC was shown in Fig. 3 (red line), the peaks at 683.94, 1479.99, and 1564.17 cm^−1^ attributable to the carboxylate group (COOH) of glycine. The presence of ammonium group NH_3_^+^ in ZrO_2_-Gly showed at 1126.19 and 2528 cm^−1^, respectively. This reflection shows that the glycine molecule exists in ZrO_2_-Gly as a zwitterionic form. Correspondingly, the peaks revealed at 847.97 and 1323.99 cm^−1^ are assigned to CCN and CH_2_ stretching groups. The existence of ZrO_2_ in the ZrO_2_-Gly was verified through the peak at 535.99 cm^−1^ and 453.97 cm^−1^, respectively. The slight shifts in the various peaks of prepared ZrO_2_ NPs and ZrO_2_-Gly could be due to the interaction between Gly and ZrO_2_ NPs.

**Figure 4:**
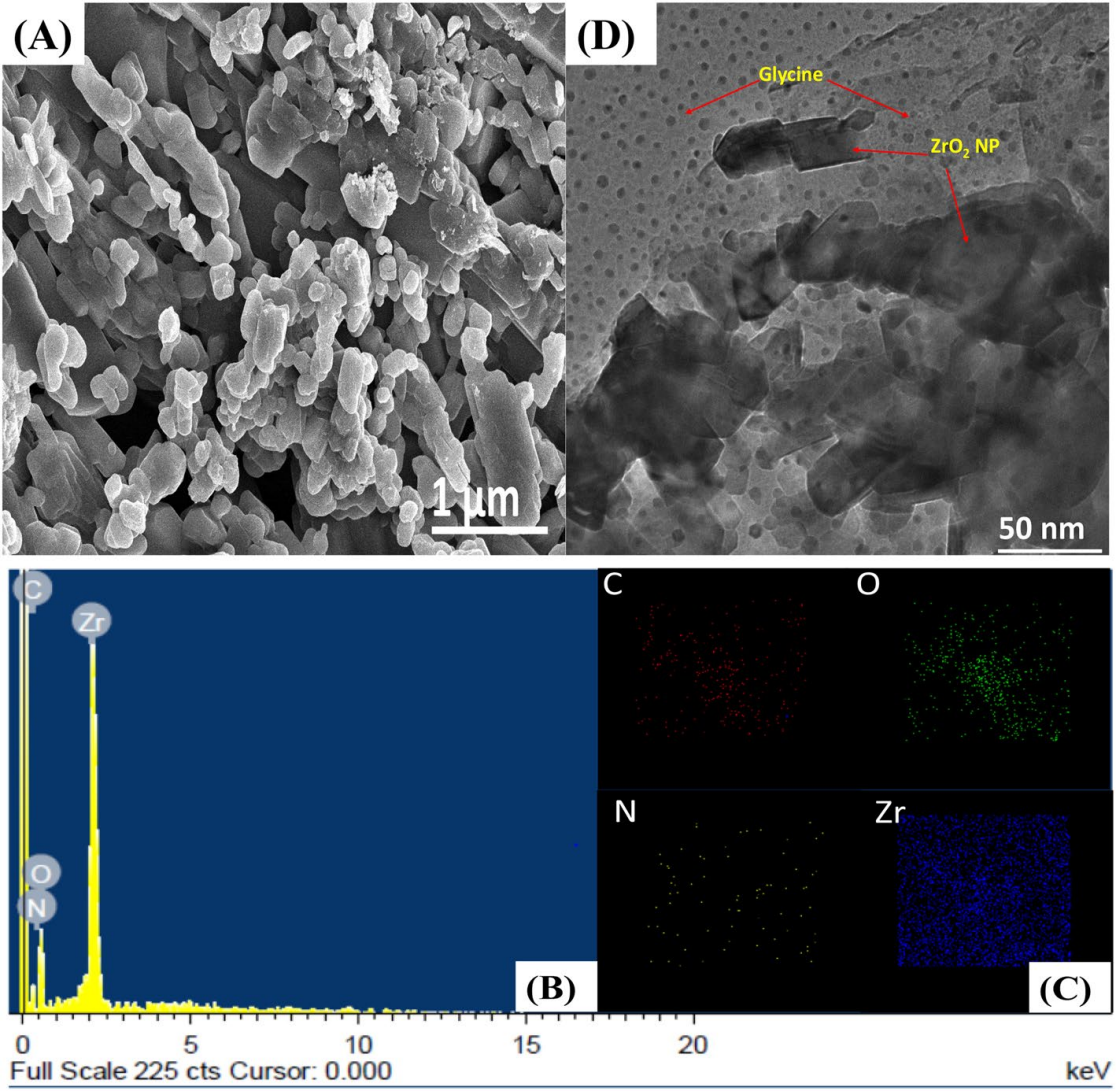
(**A**) SEM micrograph of ZrO_2_-Gly nanocomposite at 1 µm scale (**B**) EDX and (**C**) mapping (**D**) TEM micrograph at 50 nm.

An investigation of the morphology of the synthesized ZrO_2_-Gly NC was carried out using SEM and HRTEM. Figure 4A depicted a scanning electron microscope picture of ZrO_2_-Gly NC with a wrinkled microstructure of the size of 1 micron, which was interconnected by a significant number of nanoparticle connections. EDX analysis (Fig. 4B) indicated the existence of C, N, O, and Zr. Aside from that, Fig. 4C illustrates that elemental mapping evaluation gives the direct elemental distribution of the samples, which determines the homogeneous dispersion of Carbon (red), Nitrogen (yellow), Oxygen (green), and Zirconium atoms (blue) in ZrO_2_-Gly NC. Figure 4D shows the results of the TEM analysis used to evaluate the functionalization of glycine on the ZrO_2_ nanoparticle surface. Figure 4D shows that the surface of a wrinkled nanoplate morphology of ZrO_2_ nanoparticle with an average particle size of 12 nm was effectively wrapped with glycine. It has been established that ZrO_2_-Gly NC was synthesized. TEM images of ZrO_2_-Gly NC revealed the black uniform dispersion of ZrO_2_ nanoparticles implanted on the surface of the glycine molecule.

### Weight loss measurement

#### Effect of concentration and temperature

The results of weight loss measurements for MS in 1 M HCl with different concentrations of NC for 6 h at different temperatures are shown in Table 1.The experimental parameters like corrosion rate (*CR*), inhibition efficiency (*IE%*), and surface coverage (*θ*) are calculated by the following equations^31^:

**Table 1:** Corrosion parameters of MS following 6 h immersion in 1 M HCl solution in the absence and presence of different concentrations of ZrO_2_-Gly NC at different temperatures.

C (ppm)	40 °C		50 °C		60 °C		70 °C		80 °C	
	*CR* (mpy)	*IE* (%)	*CR* (mpy)	*IE* (%)	*CR* (mpy)	*IE* (%)	*CR* (mpy)	*IE* (%)	*CR* (mpy)	*IE* (%)
0	498.99 ± 2.4	–	1387.26 ± 2.8	–	2791.31 ± 2.1	–	5328 ± 2.3	–	8486.00 ± 2.7	–
50	353.02 ± 1.6	29.2 ± 0.1	930.88 ± 3.0	32.93 ± 0.2	1795.99 ± 3.2	35.68 ± 0.1	3042.98 ± 2.1	42.88 ± 0.2	5749.06 ± 3.2	32.25 ± 0.1
100	271.81 ± 1.3	45.5 ± 0.3	673.16 ± 2.1	51.49 ± 0.2	1214.77 ± 4.2	56.49 ± 0.1	2154.32 ± 1.7	59.56 ± 0.3	4420.18 ± 2.1	47.91 ± 0.2
300	169.80 ± 0.8	65.9 ± 0.2	389.26 ± 2.1	71.95 ± 0.1	678.53 ± 1.9	75.70 ± 0.4	1091.28 ± 1.9	79.51 ± 0.2	3143.66 ± 1.5	62.95 ± 0.1
500	157.04 ± 1.0	68.5 ± 0.8	326.17 ± 1.9	76.49 ± 0.4	593.29 ± 3.2	78.75 ± 0.1	1011.42 ± 2.5	81.01 ± 0.1	2244.32 ± 1.8	73.55 ± 0.7
700	154.36 ± 0.4	69.1 ± 0.1	318.79 ± 3.2	77.03 ± 0.5	579.87 ± 1.9	79.23 ± 0.2	965.78 ± 3.6	81.87 ± 0.3	2132.24 ± 1.2	74.87 ± 0.2
1000	149.66 ± 0.2	70.0 ± 0.2	304.03 ± 1.3	78.09 ± 0.2	537.59 ± 2.8	80.74 ± 0.1	951.01 ± 2.5	82.15 ± 0.2	2064.45 ± 2.3	75.67 ± 0.4

“±” shows the standard deviation of three measurements.

4 $$CR=\frac{KW}{AT\rho }$$

5 $$IE\left(\%\right)=\frac{{CR}^{0}-{CR}^{i}}{{CR}^{0}}\times 100$$

6 $$\Theta=\frac{IE(\%)}{100}$$

Here, *K*—3.45 × 10^6^ is a constant, *W*- weight loss in gram, *A*-total surface area in cm^2^, *T*—is the exposure time in h, *ρ*—density (7.86 g cm−^-3^), *IE*(%)-percent inhibition efficiency, *CR*^0^ and *CR*^i^—corrosion rates in absence and presence of inhibitor, *θ*—surface coverage.

From Table 1, it is found that with the increase of inhibitor concentration up to 500 ppm, the corrosion rate reduces appreciably, consequently, the inhibitor efficiency and the surface coverage also increase which suggests that the increase of concentration leads to an increase in the adsorption capability of the inhibitor. Above 500 ppm, the increase in ZrO_2_-Gly NC concentration does not affect the inhibition efficacies much. This phenomenon indicates that the MS surface may have been already fully covered by inhibitor molecules with the 500 ppm corrosion inhibitor in solution at 40 °C. This may be due to the saturation of adsorption of inhibitor molecules on the metal surface which exhibits a negligible increase in protection efficacies^18^.

The effect of temperature for MS corrosion in the temperature range of 40–80 °C in the test solution was also evaluated using the weight-loss method. From the data in Table 1, it can be concluded that by raising the temperature up to 70 °C, the corrosion rate increases quickly without and with the inhibitor. However, the increase in corrosion rates in the inhibited medium is less than in the blank solution which is suggestive of ZrO_2_-Gly NC adsorption on the metal surface and therefore, blocking the active corrosion sites responsible for the corrosion. This adsorption of NC leads to an increase of IE (%) with increasing the temperature and reaches to maximum up to81.01% at 500 ppm at 70 °C. This behavior is suggestive of chemisorption^32^. However, at 80 °C the inhibition efficacies decrease slightly and reached 73%. The phenomenon may be due to the desorption of inhibitor molecules on the metal surface.

#### Activation parameters

The temperature increase has a great influence on the corrosion rate, the adsorption equilibrium and kinetics. These thermodynamic activation functions were obtained through Arrhenius and the transition-state Eqs. (7) and (8)^33^:

7 $$\mathrm{log}CR=logA-\frac{Ea}{2.303RT }$$

where *A* denotes the pre-exponential factor, *T* absolute temperature in Kelvin, and *R*, the Gas Constant in JK^−1^ mol^−1^.

8 $$\nu = \frac{RT}{Nh}\mathrm{exp}\frac{\Delta S}{R}\mathrm{exp}\frac{-\Delta H}{RT}$$

where *N* is the Avogadro’s number and *h*is the Planck’s constant.

**Figure 5:**
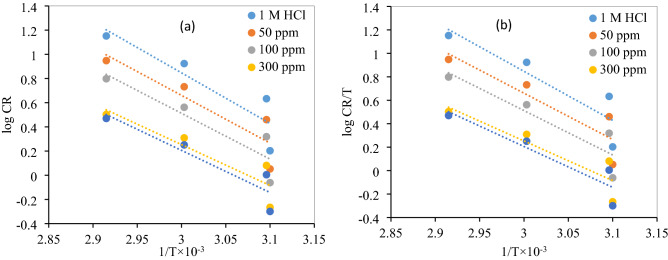
(**a**) Arrhenius, (**b**) alternative Arrhenius plots for MS in 1 M HCl in the absence and presence of different concentrations of ZrO_2_-Gly nanocomposite.

The values of activation energy (*E*_a_) calculated from the log CR vs. 1/*T* plot (Fig. 5a) are given in Table 2. The lower value of the activation energy (*E*_a_) of the process in an inhibitor’s presence when compared to that in its absence is attributed to its chemical adsorption; the physical adsorption is pronounced in the opposite case^34^. The lower value of *E*_a_ in the presence of NC compared to that in its absence and the increase of its IE (%) with temperature increase can be interpreted as an indication of chemical adsorption^35^.

**Table 2:** Activation parameters of adsorption for MS in 1 M HCl at different temperatures.

*C* (ppm)	*E*_a_	ΔH*_ads_ (KJ/mol)	ΔS*_ads_ (KJ/mol)
0	80.12	80.12	0.116
50	75.51	75.50	0.099
100	72.42	72.42	0.087
300	64.74	64.74	0.059
500	66.70	66.70	0.064

The entropy of activation (∆S*) and enthalpy of activation (∆H*) were computed using Transition State Eq. (8). From the slope (− ∆H*/2.303*R*) and intercept [log(*R*/*Nh*) + ∆S*/2.303*R*] of Fig. 5b, the values of ∆H* and ∆S* were determined respectively. All the calculated values of ∆H* and ∆S* are displayed in Table 2. The positive sign of (ΔH*) infers that the process of the mild steel dissolution is endothermic, which implies the difficulty of the mild steel corrosion in the acid solution protected with a synthesized inhibitor. The decrease in ΔS* values (Table 2) in presence of inhibitor-protected acid solutions indicate that entropy decreases on forming the transition state, which indicates an associative mechanism^36^.

#### Adsorption isotherm and thermodynamic parameters

**Figure 6:**
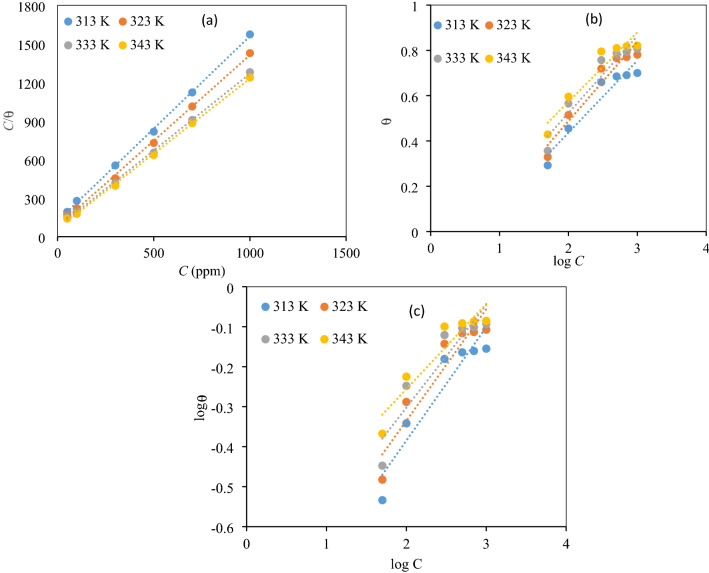
(**a**) Langmuir Adsorption, (**b**) Temkin Adsorption, (**c**) Freundlich Adsorption isotherm.

To assess the probability of an adsorption activity on the surface of the mild steel specimen, the adsorption isotherms were computed. We investigated several isothermal adsorption equations which relate the inhibitor concentration and the surface coverage. To that end we tested the isothermal adsorption models of Langmuir, Temkin, and Freundlich. In comparison to other isotherm models, the regression coefficients for Langmuir adsorption isotherm are closer to unity (Fig. 6), therefore, showing it the highest possible adsorption model for the NC molecules on the surface of mild steel specimens in the studied acidic medium. Langmuir adsorption isotherm has the form^37^:

9 $$\frac{\mathrm{C}}{\Theta}=\frac{1}{{\mathrm{K}}_{\mathrm{ads}}}+\mathrm{C}$$

where *C*, *θ*, *K*_ads_ are the concentration of inhibitor, surface coverage area, and equilibrium constant, respectively.

Herein, *K*_ads_, obtained from the intercept of the Langmuir isotherm, displays higher values, given in Table 3, meaning that NC can preferentially adsorb on the mild steel surface and thus deliver great IE values. The corresponding adsorption isotherm curves and related thermodynamic parameters such as standard Gibbs free energy of adsorption, Δ*G*°_ads_, standard adsorption enthalpy (Δ*H*°_ads_), and standard entropy of adsorption (Δ*S*°_ads_) are presented in Fig. 6 and Table 3 respectively, which are calculated using Eqs. (10)–12^38^:

10 $$\Delta G^{o}_{ads} = - RT\ln (1 \times 10^{6} K_{ads} )$$

11 $$\log K_{ads} = \frac{{ - \Delta H^{o}_{ads} }}{2.303RT} + constan t$$

12 $$\Delta G^{o}_{ads} = \Delta H^{o}_{ads} - T\Delta S^{o}_{ads}$$

where *R*: the universal gas constant, *T*: the absolute temperature and the value of 1 × 10^6^ is the concentration of water (ppm) in solution.

**Table 3:** Adsorption parameters of NC.

Langmuir				Temkin	Freundlich	Δ*H*°_ads_	Δ*S*°_ads_
*T* (°C)	*R*^2^	*K*_ads_ (ppm/mol)	Δ*G*°_ads_ (KJ/mol)	*R*^2^	*R*^2^		
40	0.9985	0.012	− 24.51	0.9182	0.8794	52.46	− 0.264
50	0.9996	0.014	− 25.56	0.9281	0.8843		− 0.101
60	0.9999	0.022	− 27.70	0.9085	0.8592		− 0.105
70	0.9998	0.231	− 35.21	0.9024	0.8746		− 0.109

**Figure 7:**
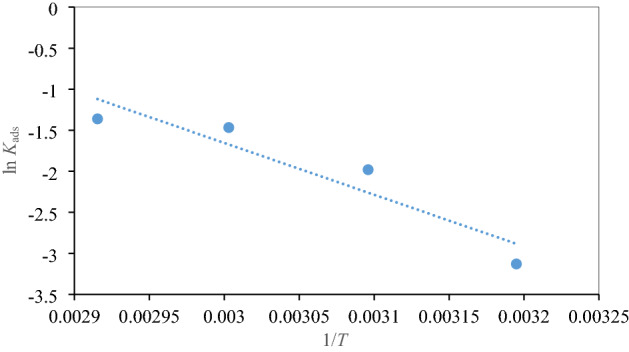
Plot of log K_ads_ vs. 1/T.

The negative values of Δ*G*°_ads_ demonstrate that the adsorption of NC is a spontaneous process, and NC can adsorb on the metal surface by forming a defensive film. The values of Δ*H*°_ads_ and Δ*S*°_ads_ calculated from Fig. 7 are given in Table 3. The calculated Δ*H*°_ads_ values with positive signs mean that the adsorption of NC molecules on steel surface is an endothermic process. Based on negative Δ*S*°_ads_ values, we can say that adherence of inhibitor molecules on the metal surface lowers the entropy^39^.

### An electrochemical impedance measurement study

**Figure 8:**
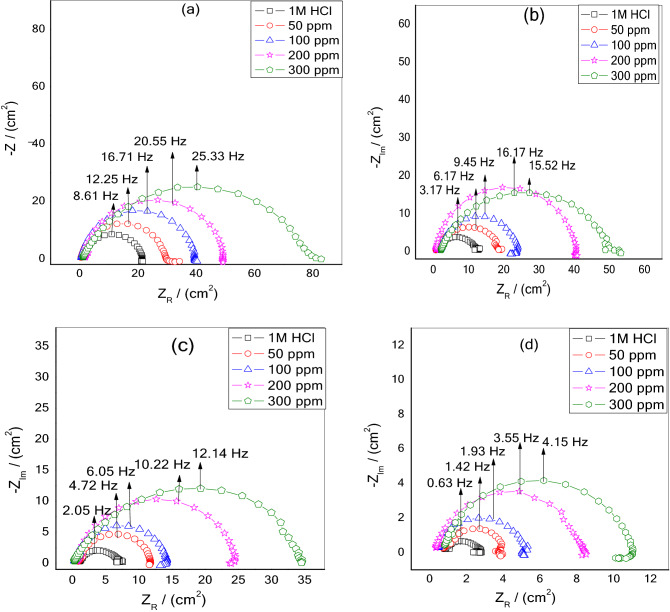
Nyquist plots for MS in 1 M HCl solution without and with various dosages of ZrO_2_-Gly NC at (**a**) 40 °C, (**b**) 50 °C, (**c**) 60 °C, (**d**) 70 °C.

**Figure 9:**
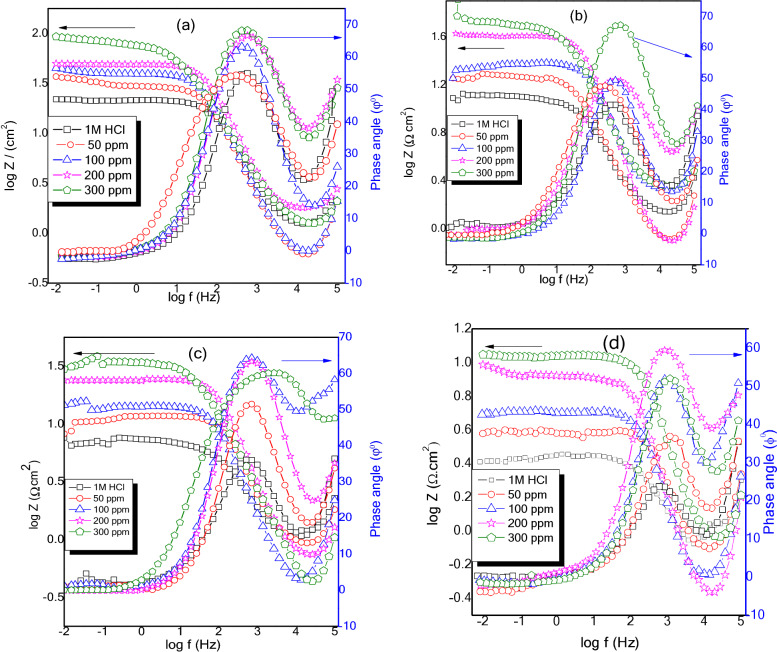
Bode plots for MS in 1 M HCl solution without and with various dosages of ZrO_2_-Gly NC at (**a**) 40 °C, (**b**) 50 °C, (**c**) 60 °C, (**d**) 70 °C.

**Figure 10:**
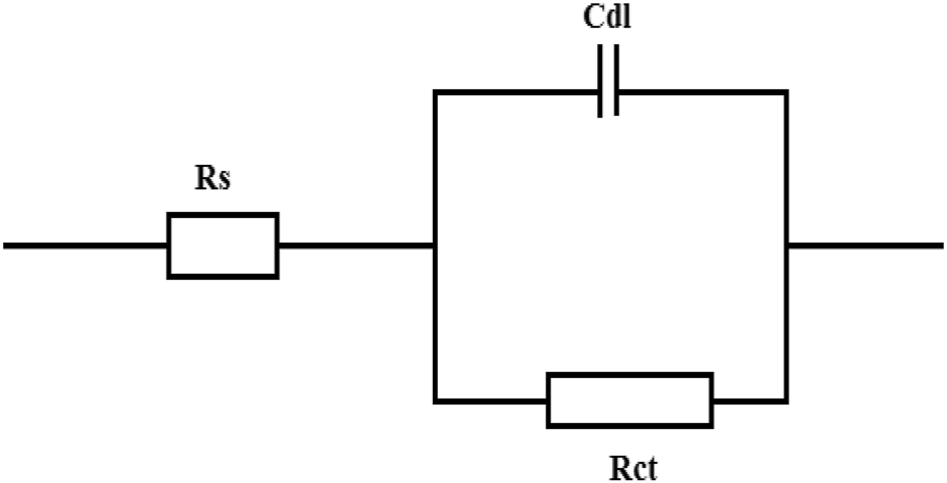
The equivalent circuit used to fit EIS spectra.

**Figure 11:**
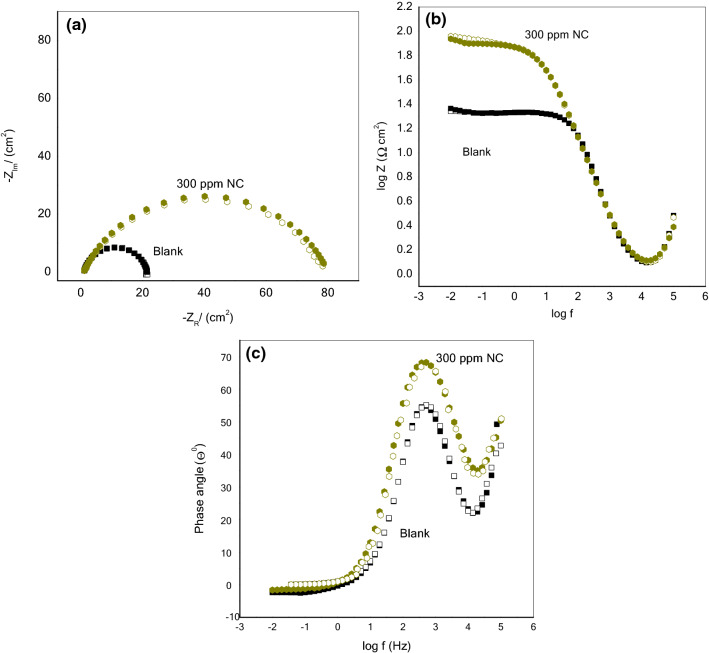
Experimental and computer fit results: (**a**) Nyquist plot; (**b**) Bode impedance plot; (**c**) Bode phase angle plot Solid and open symbol shows experimental and fitted data, respectively.

The Nyquist and Bode plots of MS in 1 M HCl without and with various concentrations of NC at (a) 40 °C, (b) 50 °C (c) 60 °C, and (d) 70 °C are depicted in Figs. 8 and 9a–d. The depressed semicircular loops of Nyquist plots were obtained at all the investigated concentrations and temperatures with the center under the real axis is typical behavior for solid-metal electrodes with frequency dispersion of the impedance data due to roughness and other in homogeneities on the sample surface^26^. Nevertheless, the spectra for various concentrations of inhibitors at all studied temperatures are identical in the shape, suggesting that there is no modification in the corrosion process^40,41^. Nonetheless, the existence of the NC in the acid solution, depending on the concentration, has an impact on the size of the semicircular curves, which implies that the introduction of NC has delayed the WE dissolution rate. Furthermore, the radius of the semicircle decreases in the absence of NC with increasing the temperature from 40 to 70 °C due to the penetration of corrosive media which facilitates the MS corrosion. In comparison to the blank, when NC was added to the corrosive solution, the impedance radius increased dramatically and continued to increase as the NC concentration increased at each temperature. As a result of this occurrence, an adsorbed layer formed on steel, which gave good corrosion resistance. Figure 10 demonstrates the equivalent circuit model which is used to fit the electrochemical impedance data. The experimental and computer fit results of the Nyquist and Bode plots for MS without and with NC are shown in Fig. 11a–c. The fitting standard of the equivalent circuit was checked by the chi-square (χ^2^) values. The small χ^2^ values i.e., 10^–4^, suggest satisfactory fitting of obtained impedance spectra to the proposed equivalent circuit. The fit results are also agreeable with the experimental data. The derived (*R*_s_, *R*_p_, *n*, *Y*_0_) and computed (*C*_dl_ and IE) parameters are displayed in Table 4. Here polarization resistance is *R*_p_, and the exponent *n*—is the phase shift representing the degree of deviation of the CPE exponent, when n = 1 it represents ideal capacitive behavior. When *n* = 0.5 it is indicative of diffusion control^42,43^ and it represents pure resistive behavior when it is equal to 0. The values of *n *approaching 1, suggest the characteristics of the pseudo capacitor are more apparent. Hence, when using capacitance (*C*) on the actual EIS, it is difficult to have a good fitting effect. Constant phase element (*CPE*) is used instead of *C* and defines CPE impedance^44^.

13 $$Z_{CPE} = \frac{{1}}{{Y_{{0}} (j\omega )^{n} }}\,$$

**Table 4:** EIS parameters for MS at different temperatures in 1 M HCl solution in the presence and absence of different concentrations of ZrO_2_-Gly NC.

*T* (°C)	*C* (ppm)	*R*_s_ (Ω cm^2^)	*R*_p_ (Ω cm^2^)	*n*	C_dl_ × 10^–5^ (F cm^−2^)	*τ*	*Χ*^2^ × 10^–4^	*IE* (%)
40	0	1.194 ± 0.001	20.4 ± 0.16	0.8872 ± 0.004	13.2	0.0184	21	–
	50	0.472 ± 0.003	29.1 ± 0.12	0.8903 ± 0.002	12.2	0.0129	14	29.86 ± 0.14
	100	0.482 ± 0.004	39.0 ± 0.27	0.9076 ± 0.001	6.9	0.0095	13	47.72 ± 0.23
	300	1.743 ± 0.002	54.0 ± 0.24	0.9086 ± 0.002	6.3	0.0077	12	62.22 ± 0.47
	500	1.218 ± 0.001	77.8 ± 0.05	0.9159 ± 0.003	6.11	0.0062	18	73.79 ± 0.41
50	0	1.115 ± 0.001	11.3 ± 0.2	0.7544 ± 0.001	12.71	0.0433	22	–
	50	0.906 ± 0.002	17.8 ± 0.07	0.8945 ± 0.002	9.05	0.0258	27	36.56 ± 0.21
	100	2.017 ± 0.004	22.3 ± 0.09	0.8953 ± 0.002	6.93	0.0168	65	49.02 ± 0.19
	300	0.630 ± 0.003	40.0 ± 0.18	0.8998 ± 0.001	6.12	0.0094	34	71.64 ± 0.25
	500	1.977 ± 0.002	49.2 ± 0.22	0.9104 ± 0.003	5.6	0.0102	21	76.93 ± 0.34
60	0	0.795 ± 0.001	6.3 ± 0.02	0.7286 ± 0.002	14.8	0.0776	12	–
	50	0.902 ± 0.004	11.1 ± 0.05	0.8966 ± 0.004	8.26	0.0337	11	42.69 ± 0.21
	100	0.253 ± 0.005	14.1 ± 0.06	0.9052 ± 0.006	6.58	0.0263	19	54.96 ± 0.25
	300	0.647 ± 0.001	24.1 ± 0.10	0.9196 ± 0.002	6.01	0.0155	10	73.75 ± 0.30
	500	0.464 ± 0.001	35.2 ± 0.15	0.9202 ± 0.001	5.67	0.0131	9	82.00 ± 0.42
70	0	0.944 ± 0.002	1.8 ± 0.11	0.7078 ± 0.004	26.0	0.2527	4	–
	50	0.705 ± 0.003	3.4 ± 0.12	0.9031 ± 0.003	8.12	0.1121	6	46.93 ± 0.23
	100	0.347 ± 0.001	4.9 ± 0.14	0.9160 ± 0.002	6.27	0.0825	8	63.33 ± 0.18
	300	0.362 ± 0.001	7.8 ± 0.16	0.9263 ± 0.001	5.84	0.0448	9	77.08 ± 0.15
	500	0.744 ± 0.002	10.3 ± 0.12	0.9378 ± 0.001	5.57	0.0383	12	82.62 ± 0.34

“±” shows the standard deviation of three measurements.

Here, *CPE *is called a dummy capacitor, *n* represents the degree to which the dummy capacitance deviates from the pure capacitance. In the present study the *CPE *exponents (*n*) are in the range of 0.890 to 0.9159 at 40 °C, 0.894 to 0.910 at 50 °C, 0.896 to 0.920 at 60 °C and 0.90.3 to 0.937at 70 °C and these values are more than the blank solution (0.88721, 0.75436, 0.72865, 0.70781 at 40, 50, 60 and 70 °C, respectively). The increase in values *n* implies that the NC reinforce the homogeneity rate of MS by forming an adsorptive film over the steel surface.

The relation time constant (*τ*)^45^ of the adsorption process is calculated following Eq. (14)

14 $$\tau =\frac{1}{2\pi {f}_{max}}$$

The *τ *decreased at all studied temperatures i.e., 40, 50, 60, and 70 °C respectively in presence of NC as compared to blank, indicating that charging and discharge levels for metal-solution interfaces decreased significantly^46^.

Moreover, the values of *R*_p_ and *C*_dl_ as could be seen in Table 2 vary in the opposite direction with NC concentrations, i.e., *R*_*p*_ increase and *C*_dl_ decrease with an increase in inhibitor amount. The pattern found is in line with those identified by other researchers^47^. This finding demonstrates that the NC develops a protective film that prevents the dissolution of the mild steel.

It is seen that the values of *R*_p_ in the inhibitor-containing solution are much larger than that in inhibitor-free solution, especially at 500 ppm. For example, the value of *R*_p_ increases from 20.4 to 29.1 Ω cm^2^ after adding 50 ppm, and the corresponding IE (%) reaches 29.86%. By increasing the concentration of NC to 500 ppm, the value of *R*_p_ increases to 77.8 Ω cm^2^ and the corresponding IE (%) reaches 73.79%.

**Figure 12:**
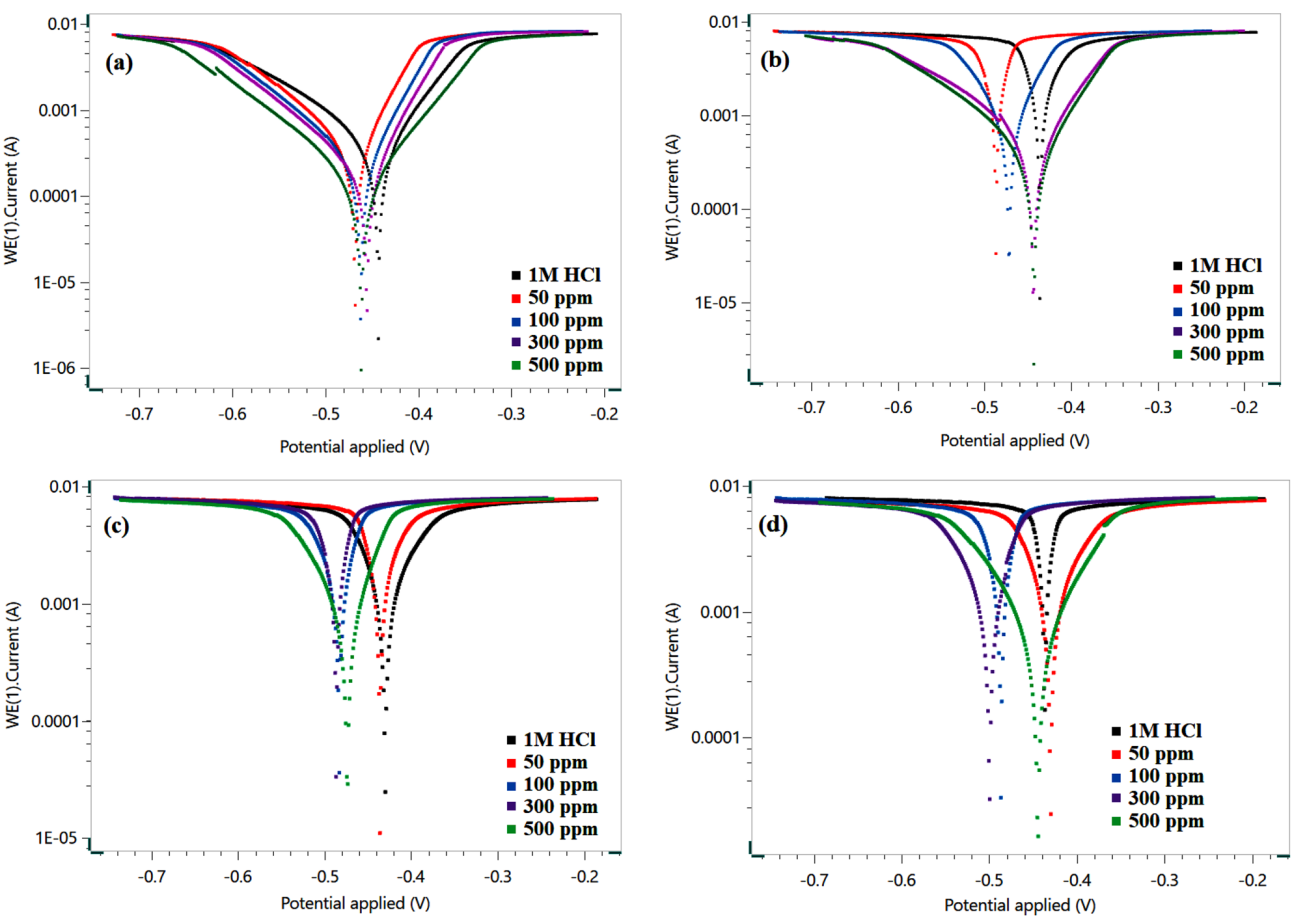
PDP plots for MS in 1 M HCl solution without and with various dosages of ZrO_2_-Gly NC at (**a**) 40 °C, (**b**) 50 °C, (**c**) 60 °C and (**d**) 70 °C.

Figure 12a–d shows corresponding Bode plots for MS at different temperatures.In the Bode diagrams, log [Z] and phase angle (*ϕ*°) are plotted against log f. The Bode graphs illustrate that at each concentration of the NC, there is just one phase maxima suggesting that corrosion process is occurring through one-time constant^48^. For pure capacitive behavior, a linear relationship between log f and log Z should be produced with a slope of − 1. The corrosion prevention properties of studied NC are confirmed by the increase in the absolute value of the impedance at low frequencies, which is due to the formation of an inhibitory film that prevents MS to come in the contact with the corrosive solution.The slope close to − 1 and phase angle (*θ*)close to 90° suggests the capacitive behavior and the modulus has increased as compared with the blank with the addition of ZrO_2_-Gly NC and thus suggest that the perturbations induced by the frequency changes on the metal electrode result in the protection of iron^49^.

### Potentiodynamic polarization studies

To gain more insight into the effectiveness and adsorption process of NC at higher temperatures, the polarization tests were also conducted on MS in the studied HCl solution without and with an inhibitor. The test was performed at temperatures of 40, 50, 60, and 70 °C, and polarization curves are shown in Fig. 12a–d. The potentiodynamic polarization parameters such as corrosion potential (*E*_corr_), corrosion current density (*i*_corr_), and anodic and cathodic Tafel slope (*β*_a_ and *β*_c_) are presented in Table 5. The *E*_corr_ values given in the Table 5 confirm the mixed type of behavior of NC. The corrosion potential of inhibited systems is displaced toward a more negative direction relative to the blank. The negative displacement of *E*_corr_ values can be successfully explained by the fact that NC produces a more beneficial effect on the cathodic type of reaction i.e., H^+^ ion reduction than that of the anodic reaction i.e., MS dissolution^50,51^. This implies that the protective inhibitor film formed on the MS surface suppresses the transfer of H^+^ ions to the cathodic site of mild steel surface and hereby decreases the evolution rate of H_2_ gas. As seen in Fig. 12, a decline in the cathodic and anodic current density values is observed in rising NC concentrations. This phenomenon can be explained by the adsorption of NC to active centers in both cathodic and anodic regions. The similar trend of Tafel curves could be observed from different temperatures.

**Table 5:** Potentiodynamic polarization parameters for MS at 30 °C in 1 M HCl solution in the presence and absence of different concentrations of ZrO_2_-Gly NC.

*C* (ppm)	T (°C)	*E*_corr_ (mV vs. Ag/AgCl)	*β*_a_ (mV dec^−1^)	*− β*_c_ (mV dec^−1^)	i_corr_ (μA cm^−2^)	*CR* (mm/year)	*IE* (%)
0	40	− 431.9 ± 2.2	95.8 ± 0.42	168.5 ± 0.84	522.5 ± 2.6	6.07 ± 0.03	–
50		− 465.8 ± 2.3	62.6 ± 0.23	122.5 ± 0.62	406.7 ± 2.2	4.72 ± 0.01	22.22 ± 0.1
100		− 459.9 ± 2.1	62.8 ± 0.25	115.3 ± 0.34	267.0 ± 1.9	3.10 ± 0.02	48.94 ± 0.2
300		− 451.3 ± 1.9	57.9 ± 0.65	119.9 ± 0.56	203.3 ± 2.6	2.36 ± 0.02	61.11 ± 0.3
500		− 453.2 ± 1.1	54.3 ± 0.21	116.8 ± 0.67	158.6 ± 1.2	1.70 ± 0.01	69.68 ± 0.3
0	50	− 437.6 ± 2.1	368.3 ± 0.32	99.2 ± 0.71	2737.2 ± 11.1	66.66 ± 0.33	–
50		− 486.2 ± 2.2	42.6 ± 0.45	57.8 ± 0.22	2179.7 ± 12.1	25.33 ± 0.12	20.36 ± 0.2
100		− 470.4 ± 2.5	80.9 ± 0.41	99.9 ± 0.27	1216.2 ± 1.9	14.13 ± 0.06	54.95 ± 0.1
300		− 436.7 ± 1.8	110.6 ± 0.34	199.7 ± 0.34	778.7 ± 3.4	9.04 ± 0.040	71.15 ± 0.4
500		− 431.9 ± 1.4	95.8 ± 0.21	168.5 ± 0.56	522.5 ± 5.4	6.07 ± 0.03	80.64 ± 0.2
0	60	− 429.5 ± 1.3	948.8 ± 0.65	240.4 ± 0.61	8133.1 ± 2.9	94.50 ± 0.50	–
50		− 435.5 ± 2.1	329.8 ± 0.34	98.0 ± 0.23	5538.2 ± 28.3	64.35 ± 0.32	31.90 ± 0.2
100		− 482.5 ± 2.5	721.0 ± 0.54	138.1 ± 0.18	3061.5 ± 25.2	35.57 ± 0.18	62.34 ± 0.1
300		− 486.1 ± 2.2	51.8 ± 0.23	73.5 ± 0.42	2738.7 ± 20.2	31.82 ± 0.24	66.31 ± 0.3
500		− 473.4 ± 1.7	731.0 ± 0.18	106.6 ± 0.18	1142 ± 18.9	13.27 ± 0.17	85.95 ± 0.4
0	70	− 436.2 ± 1.2	337.8 ± 0.15	138.7 ± 0.52	15,213 ± 23.1	176.77 ± 0.78	–
50		− 429.5 ± 1.8	948.8 ± 0.34	240.5 ± 0.36	8133.1 ± 17.5	94.51 ± 0.65	46.54 ± 0.2
100		− 486.1 ± 1.9	65.2 ± 0.45	115.1 ± 0.38	3776.1 ± 12.3	43.87 ± 0.87	75.17 ± 0.1
300		− 499.3 ± 2.3	97.6 ± 0.21	194.1 ± 0.32	2825.9 ± 14.6	32.83 ± 0.43	81.42 ± 0.3
500		− 444.0 ± 2.0	119.4 ± 0.10	122.6 ± 0.81	954.571 ± 17.8	11.09 ± 0.21	93.72 ± 0.4

“±” shows the standard deviation of three measurements.

Further, as the temperature increases, the corrosion rate increases both in the HCl solution without and with the inhibitor but the increase in corrosion rate is less as compared to blank at each concentration. Table 5 shows that *i*_corr_ values increases as temperature rises for the blank solution. Analysis of the results in Table 4 indicates the pronounced increase in *i*_corr_ values for a blank solution as the temperature rises. However, in the presence of NC molecules, the *i*_corr_ of MS decreases at any given temperature at all studied concentrations due to the increase of the surface coverage degree^52^. The percentage of inhibitor efficiency (IE%) is calculated from Eq. (1) and tabulated in Table 5. The inhibition efficiency increased with temperature according to the following order: 70 °C > 60 °C > 50 °C > 40 °C. The higher inhibition efficiency at 70 °C may be attributed to the higher electronic density of the functional groups resulting in the formation of a more adsorbent, and as a result, it caused the highest inhibition. This represents a greater chemical adsorption contribution suggesting that the bond between the inhibitor molecules and the metal surface is more likely to be electron sharing. When the polarization potential continues to increase, it can be observed that the curves are close to coinciding. This is likely due to the rapid dissolution of MS at the strong polarization potential, causing the desorption of NC from the metal surface^53,54^.

### Contact angle measurement

**Figure 13:**
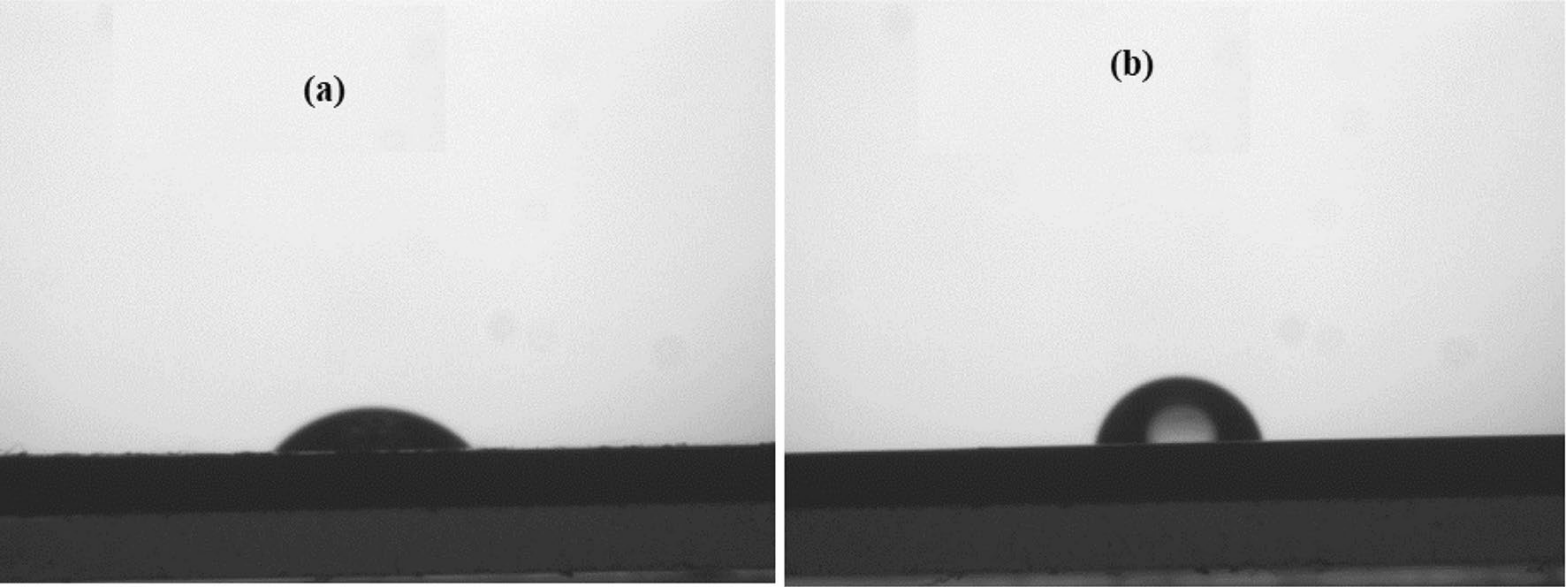
Contact angle measurement (**a**) MS immersed in acidic media, (**b**) MS immersed in acidic media containing ZrO_2_-Gly NC.

Contact angle measurement is a normal tool to understand the wetting capacity. A contact angle less than 90° usually indicates that wetting of the surface is very favorable, and the fluid will spread over a large area of the surface. Contact angle greater than 90° generally mean that wetting of the surface is unfavorable, so the fluid will minimize contact with the surface and form a compact liquid droplet^55^. In the present study, the water contact angle (*θ*_water_) value of uninhibited MS surface reached 44.98° suggesting a greater affinity of MS surface towards the corrosive environment (Fig. 13a) while NC inhibited MS surface showed the *θ*_water_ value at 72.2° (Fig. 13b). As compared to the uninhibited surface, higher water contact angle values on the NC adsorbed MS surface indicate that NC intensified the hydrophobicity means the metal surface is now less prone to water^56^. The hydrophobicity of NC molecule slows down the penetration of the water-soluble corrosive components at the MS surface^57^.

### Scanning electron microscopy (SEM)-energy dispersive X-ray spectroscopy (EDS) study

**Figure 14:**
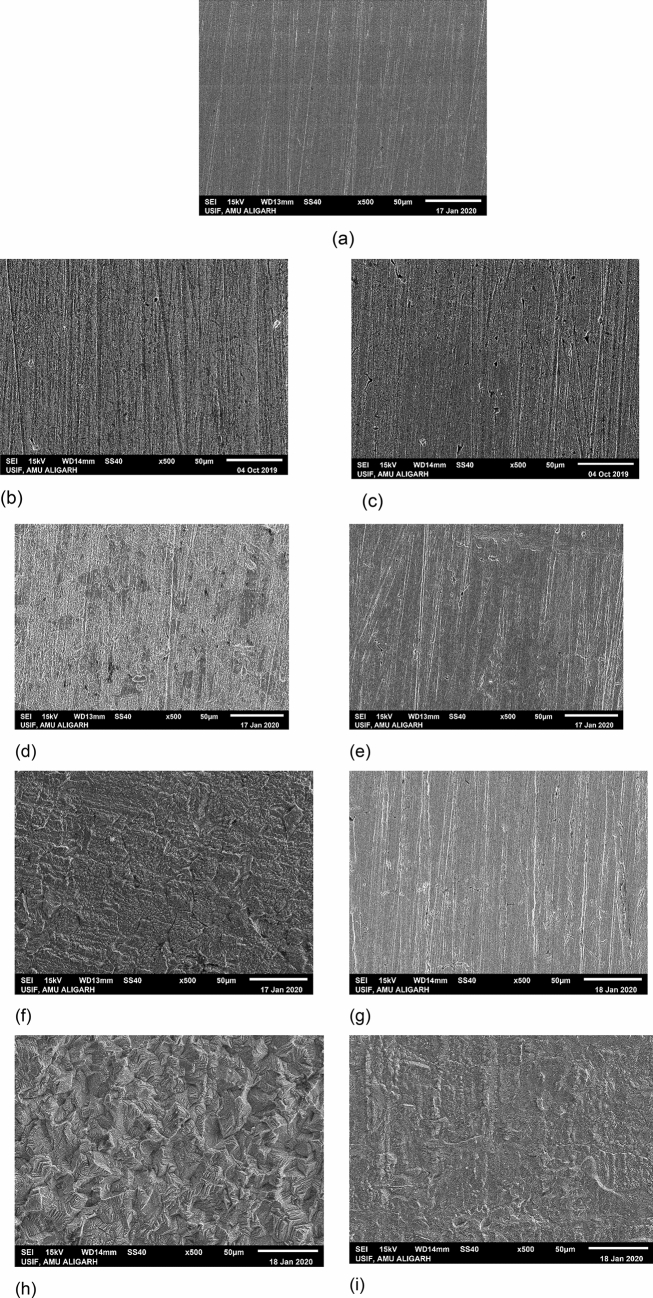
SEM photomicrographs (**a**) polished MS surface (**b**) MS surface immersed in uninhibited acid at (**b**) 40 °C, (**d**) 50 °C, (**f**) 60 °C, (**h**) 70 °C, (**c**) MS surface immersed in 500 ppm ZrO_2_-Gly NC inhibited acid at (**c**) 40 °C, (**e**) 50 °C, (**g**) 60 °C, (**i**) 70 °C.

Figure 14 illustrates the SEM images of test coupons. Figure 14a shows the topology of the MS plain surface without immersion. It shows a defect-free surface with some scratches arising out of polishing. Figure 14b, d, f, and h shows the SEM micrographs of the corroded surface formed in 1 M HCl solution at different temperatures (40–70 °C). The surface at 40 °C becomes rough and uneven, and some pits were also observed (Fig. 14b) due to uncontrolled attack of aggressive media on the metal surface leading to the dissolution of metal. Furthermore, as the temperature rises from 40 to 70 °C (Fig. 13d,f,h), the surface becomes increasingly rough due to increased corrosion rates and is therefore severely damaged. In contrast, the surface becomes smooth and uniform after 6 h immersion in 1 M HCl with 500 ppm NC (Fig. 14c) at 40 °C. The further increase in temperature leads to increase protection capabilities of studied NC which are exhibited by the smooth surface of mild steel (Fig. 14e,g,i) and the highest inhibition capabilities were seen at the temperature of 70 °C (Fig. 14i). The surface examination of the test coupons by SEM studies, therefore, validates the capability of NC to protect the metal surface appreciably at elevated temperatures.

**Figure 15:**
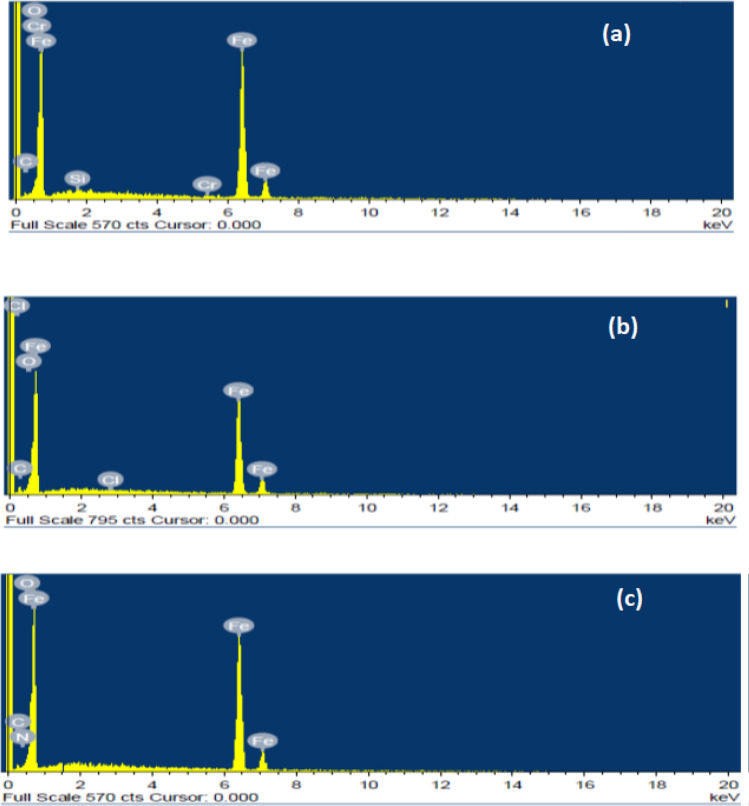
EDS photomicrographs (**a**) polished MS surface, (**b**) MS surface immersed in uninhibited acid, (**c**) MS surface immersed in 500 ppm ZrO_2_-Gly NC inhibited acid.

Figure 15a shows the EDS profile of MS plain surface without immersion which shows elemental composition in wt% as Fe-93.86, O-1.69, C-3.89, Cr-0.22, Si-0.34 pointing towards fewer oxides and more iron content in the elemental state. Figure 15b shows the EDS profile of MS with an immersion time of 6 h in HCl without NC with an elemental composition in wt. % as Fe-85.13, O-7.73, C-6.99, and Cl-0.15 indicating oxides formation with low elemental iron, and the surface is markedly corroded. NC inhibited surface (Fig. 14c) shows mild steel surface with its elemental wt% composition as Fe-92.0, O-4.70, and N-2.32 signifying less tendency of iron oxides formation and retention of the original iron.

### Inhibition mechanism

**Figure 16:**
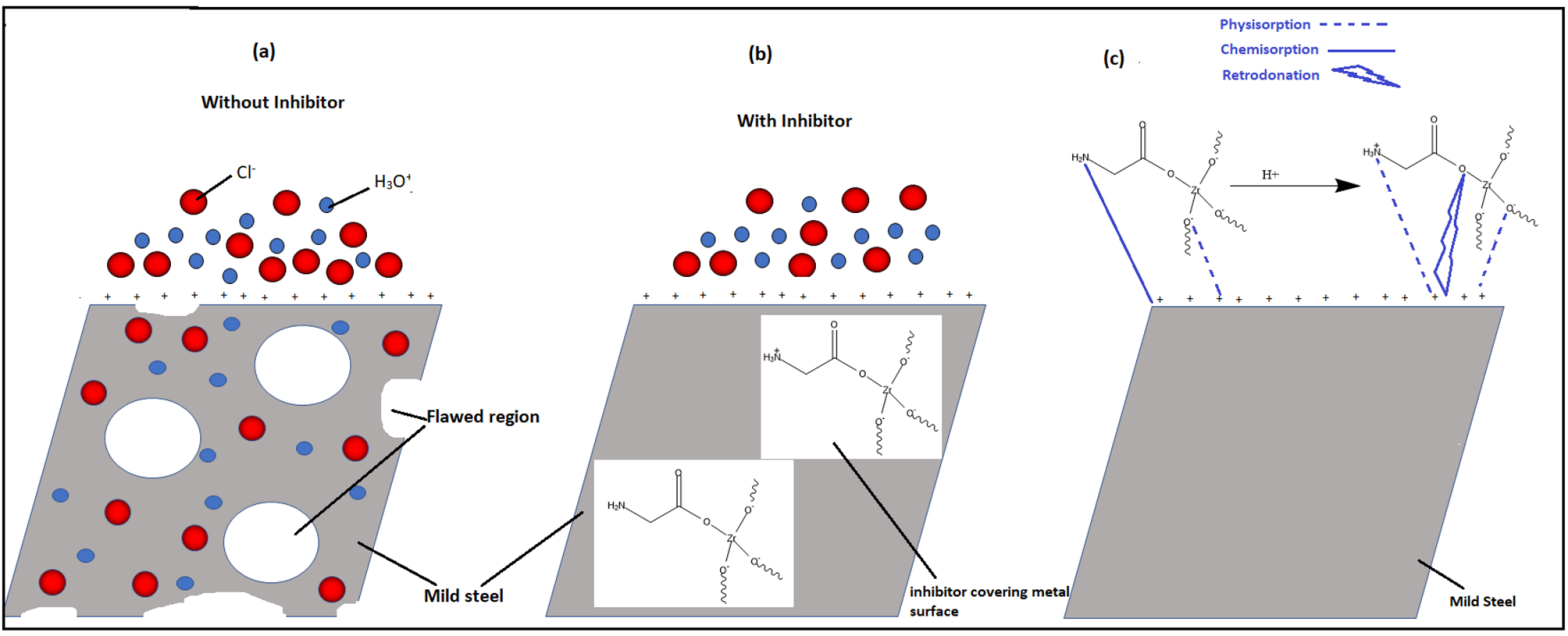
Possible adsorption mechanism of NC on the metal surface.

Amino acids strongly bind to the ZrO_2_ surface in the dissociative adsorption configuration mainly because of the covalent interaction between the oxygen atoms of the amino acids and the Zr atoms. Glycine strongly binds to Zr6f ion either via the amino (-NH_2_) or the carboxyl group (-COOH). However, the most stable binding mode for glycine occurred via the interaction of the two oxygen atoms of the carboxylic group with two Zr6f sites. According to the adsorption isotherm results as mentioned above, the existence of both chemical and physical interaction is confirmed between ZrO_2_-Gly NC and the steel surface. Mild steel is charged positively on its surface in the presence of acidic media^58,59^ and therefore, chloride ions from aggressive media get adsorbed on the metal surface (Fig. 16a). NC is present on the surface in both protonated and neutral forms. ZrO_2_-Gly NC molecules are protonated in the acid solutions through their heteroatoms. These cations are electrostatically drawn onto the MS surface already hydrated with Cl^−^ (HCl medium) (Fig. 16b). At the same time, NC can be chemically adsorbed on the steel surface on account of coordination bonds formed by transfer of lone pair electrons of N‐containing side groups to Fe atoms, as well as back‐donation of electrons between Fe atoms and –COOH group (Fig. 16c). Based on above points, we hypothesized that NC could form a protective layer and lead to a change in the reduction in corrosion rates.

## Conclusion

From the chemical, electrochemical and surface studies obtained throughout the paper, we can conclude that:

1. The prepared NC act as a successful corrosion inhibitor for MS in 1 M HCl solution.
2. Their inhibition effects increase with increases in both concentration and temperature and reached upto 81.01% at 70 °C and then decrease slightly at 80 °C reaching upto 73.55% at 500 ppm. Above 500 ppm a slight increase in inhibition efficacies were observed at all temperatures.
3. Tafel polarization curves proved that NC act as a mixed-type inhibitor with a more beneficial effect on the cathodic type of reaction.
4. EIS results showed that the polarization resistance (R_p_) increases, and double-layer capacitance (C_dl_) decreases in the presence of the NC, which suggests its adsorption on the steel surface.
5. The adsorption process of NC on steel surface is highly strengthened due to chemisorption and follows the Langmuir adsorption isotherm model.
6. SEM images exhibited that surface roughness of MS was note worthily diminished in presence of NC indicating obvious proof of its adsorption on a metal substrate and sufficient safeguards in 1 M HCl.

## Competing interests

The authors declare no competing interests.

## Data Availability

The data sets used and/or analyzed during the current study are available from the corresponding author on reasonable request.
